# Epidemiological analysis of bovine ephemeral fever in 2012–2013 in the subtropical islands of Japan

**DOI:** 10.1186/s12917-016-0673-0

**Published:** 2016-03-09

**Authors:** Yoko Hayama, Sachiko Moriguchi, Tohru Yanase, Moemi Suzuki, Tsuyoshi Niwa, Kazufumi Ikemiyagi, Yoshiki Nitta, Takehisa Yamamoto, Sota Kobayashi, Kiyokazu Murai, Toshiyuki Tsutsui

**Affiliations:** Viral Disease and Epidemiology Research Division, National Institute of Animal Health, National Agriculture and Food Research Organization, 3-1-5 Kannondai, Tsukuba, Ibaraki 305-0856 Japan; Department of Environmental Science Graduate School of Science and Technology, Niigata University, Niigata, Japan; Kyushu Research Station, National Institute of Animal Health, National Agriculture and Food Research Organization, Kagoshima, Japan; Yaeyama Livestock Hygiene Service Center, Okinawa Prefectural Government, Okinawa, Japan; Okinawa Prefectural Institute of Animal Health, Okinawa, Japan

**Keywords:** Arboviral disease, Atmospheric dispersal modeling, Bovine ephemeral fever, Conditional autoregressive model, *Culicoides* biting midges, Epidemiology, Japan, Mosquito, Spatial analysis, Windborne spread

## Abstract

**Background:**

Bovine ephemeral fever (BEF) is a febrile disease of cattle that is transmitted by arthropod vectors such as mosquitoes and *Culicoides* biting midges. An outbreak of BEF recently occurred in Ishigaki Island and surrounding islands that are located southwest of Japan. In this study, an epidemiological analysis was conducted to understand the temporal and spatial characteristics of the outbreak. Factors associated with the disease spread within Ishigaki Island were investigated by hierarchical Bayesian models. The possibility of between-island transmission by windborne vectors and transmission by long-distance migration of infected vectors were examined using atmospheric dispersion models.

**Results:**

In September 2012, the first case of the disease was detected in the western part of Ishigaki Island. In 1 month, it had rapidly spread to the southern part of the island and to surrounding islands, and led to 225 suspected cases of BEF during the outbreak. The dispersion model demonstrated the high possibility of between-island transmission by wind. Spatial analysis showed that paddy fields, farmlands, and slope gradients had a significant impact on the 1-km cell-level incidence risk. These factors may have influenced the habitats and movements of the vectors with regard to the spread of BEF. A plausible incursion event of infected vectors from Southeast Asia to Ishigaki Island was estimated to have occurred at the end of August.

**Conclusion:**

This study revealed that the condition of a terrain and land use significantly influenced disease transmission. These factors are important in assessing favorable environments for related vectors. The results of the dispersion model indicated the likely transmission of the infected vectors by wind on the local scale and on the long-distance scale. These findings would be helpful for developing a surveillance program and developing preventive measures against BEF.

## Background

Bovine ephemeral fever (BEF) is an arboviral (short for “arthropod-borne viral”) disease of cattle and water buffalos that is caused by the BEF virus (BEFV), which is a member of the genus *Ephemerovirus* in the family *Rhabdoviridae*. Bovine ephemeral fever is geographically distributed from tropical to temperate zones such as parts of Australia, Asia, the Middle East, and Africa [1–6]. The characteristic clinical signs of BEF are the sudden onset of a high fever, anorexia, depression, ocular and nasal discharge, salivation, muscle stiffness, lameness, ruminal stasis, sternal recumbency, and other inflammatory responses [1, 2]. The disease can cause severe economic impact through the reduction of milk production in dairy cattle and the loss of condition in beef cattle. The symptoms of BEF usually subside within a few days [2]. Hematophagous arthropod vectors of BEF have not been clearly defined [7], although mosquitoes and *Culicoides* biting midges are possible vectors that transmit the BEFV [1, 2, 7].

In Japan, the domestic animal infectious disease control law designates BEF as a notifiable infectious disease. The disease has not occurred for more than 20 years in the mainland of Japan [8]. The recent occurrences of BEF occurred only in Okinawa, which is a subtropical archipelago in the southwestern-most region of Japan. In 1989 and 2001 in Okinawa, large-scale outbreaks of BEF affected more than 1000 animals, and in 2002 and 2004 small-scale outbreaks occurred [8]. In 2012, an outbreak of BEF occurred in Okinawa after an 8-year absence [9]. This outbreak occurred in the Yaeyama Islands, which form an archipelago approximately 400 km southwest of the Okinawa Islands and 280 km east of Taiwan. During this outbreak, BEF was confirmed in Ishigaki Island (223 km^2^), Iriomote Island (289 km^2^), Kohama Island (7.8 km^2^), and Kuroshima Island (10 km^2^). In Ishigaki Island, in particular, the disease spread widely and affected more than 200 farms of 523 farms at risk.

In previous studies, BEFVs isolated in Japan were closely related to the Taiwanese strains, based on phylogenetic analysis of the glycoprotein gene. [10–12]. The BEFV isolated in 2012 in Japan was also classified in the same cluster of strains isolated in Taiwan and China in 1996–2004. The BEFV isolated during the large BEF epidemic in Taiwan in 2012 was also included in the same cluster and closely related to the strain isolated in China in 2002 [9, 13]. In addition, the recent outbreaks of BEF in the Yaeyama Islands were synchronized with the large epidemics in Taiwan, which suggested an epidemiological linkage between these regions [9, 10, 13]. These findings suggested that BEFVs were introduced from a gene pool in a lower latitude region and widely spread among East Asian countries during each epidemic. The incursion of BEFVs was supposedly caused by long-distance transmission by wind from lower latitude regions (which include southern China) to Taiwan and/or Japan [10].

The long-distance transport of insects by wind is considered a cause of outbreaks of agricultural pests or insect-borne plant and animal disease [14, 15]. Wind can disperse *Culicoides* biting midges, a major vector of arboviruses in ruminants, up to hundreds of kilometers [16], although the insect’s usual movements are short and range from a few hundred meters to less than 2 km [17]. The possibility of long-distance distribution of biting midges has been well investigated in outbreaks of bluetongue in Europe and Australia by using atmospheric dispersion models to estimate the potential sources of the disease and to simulate the dispersion of biting midges from source sites [18–24]. Flights of more than 100 km by wind have been recorded for several species of mosquitoes, although flight activity varies greatly between species [25, 26]. A past outbreak of BEF in Australia reportedly spread nearly 2000 km over land along with the window direction [27].

Environmental and climatic conditions in a local area affect vector habitats and influence the spread of an arbovirus disease. Mosquitoes and *Culicoides* biting midges use a wide variety of aquatic and semiaquatic environments for breeding and growth; however, wetlands such as paddy fields and bogs provide a favorable habitat for some vector species of arboviruses [25, 28, 29]. Temperature, humidity, and rainfall reportedly affect adult survivorship and egg development in these vectors [28, 30, 31]. After the outbreaks of bluetongue in Europe, climatic conditions, environmental conditions such as landscape and farm husbandry, and host availability have been proven to influence biting midges abundance [32–36]. These investigations revealed a positive relationship between biting midges abundance and pasture cover [32], prairies and woodlands [36], cattle density, and dense vegetation [34, 35]. In addition, a relationship has been demonstrated between the spatial distribution of confirmed cases of West Nile virus (which is transmitted by mosquitoes) and landscapes such as shrubland, row crops, and wetland [37]. Topographical and climatic conditions also affect the habitats of mosquitoes [38].

In this study, an epidemiological analysis of the BEF outbreak in 2012 in Japan was conducted. First, the spatial and temporal features of the BEF epidemic were described. In this paper, we used an atmospheric dispersion model to examine the likelihood that windborne infected vectors were responsible for between-island transmission of the disease. Second, to clarify the risk factors associated with the disease spread, a spatial analysis was conducted to investigate the influence of landscape and topographical conditions on the disease spread within Ishigaki Island, where most BEF cases were confirmed. Third, the hypothesis that the long-distance dispersion of infected vectors occurred from surrounding Asian regions to Yaeyama was examined. Plausible incursion events and possible source sites of infected vectors were estimated using an atmospheric dispersion model.

## Methods

### Data and descriptive analysis

In this study, an “infected farm” was defined as a farm that had been notified of a BEF-suspected case by a farmer or by a private veterinarian and the disease was subsequently diagnosed by a local veterinary officer. For the first case on each island and some notified cases on a farm located distant from other infected farms, blood samples were collected from cattle and confirmed by virus isolation and a viral neutralization test conducted at the Okinawa Prefectural Institute of Animal Health (Okinawa, Japan). Other notified cases were diagnosed based on the clinical onset of the disease and the epidemiological situations.

Information on the infected farms, date of notification, details of clinical onset, farm type, farm size, and farm location (longitude and latitude) were collected by local veterinary officers. These data were used for spatial and temporal descriptive analysis. For Ishigaki Island, information on the noninfected farms such as farm type, farm size, and farm location (i.e., longitude and latitude) was also organized by local veterinary officers for the spatial analysis. These farm information was collected as a part of epidemiological investigation based on the domestic animal infectious disease control law and was permitted to be used in this study from the animal health authority of Okinawa Prefecture. This study did not include any experimental research on animals. Therefore, approval by an ethical committee was not required. Clinical inspection and blood sampling for confirmation diagnosis were performed by local veterinary officers in accordance with the law. All maps in this study were generated using Arc GIS 10 (Esri, Redlands, CA).

### Atmospheric dispersion model

Two types of atmospheric dispersion models were applied to examine the possibility of between-island transmission by windborne infected vectors and to estimate the potential source site of infected vectors. The Hybrid Single Particle Lagrangian Integrated Trajectory (HYSPLIT) Model (National Oceanic and Atmospheric Administration [NOAA] Air Resources Laboratory, College Park, MD) is a computer model that is used to compute air parcel trajectories and the dispersion or deposition of atmospheric pollutants or particles (e.g. insects) [39]. The HYSPLIT (version 4) model has been previously used to model long-distance dispersal of *Culicoides* biting midges [18, 24, 40, 41] and other insects [42]. An overview of the HYSPLT model is described in detail in a paper by Draxler and Hess [43, 44]. In this study, back-trajectory analysis was conducted using the HYSPLIT model to estimate the potential source sites of infected vectors. The meteorological dataset used as input for the model was the Global Data Assimilation System (GDAS), which is available in 3-h intervals with a global resolution of 1° latitude/longitude (approximately 100 km^2^).

The Meteorological Data Explorer (METEX) is also an atmospheric dispersion model used to calculate air trajectory; it was developed at the Centre for Global Environmental Research (CGER) in Tsukuba, Japan [45] and was used to examine the possibility of the between-island dispersion of infected vectors by wind. Because the distance between Ishigaki Island and neighboring islands is approximately 15 km, we believe it is plausible to examine the effect of local wind in relation to the transport of infected vectors. In comparison to the HYSPLIT model, the METEX model calculates trajectories by using a more detailed meteorological dataset with a resolution of 0.1 °latitude and 0.15 °longitude (approximately 10 km^2^), based on grid point value data from the Japan Meteorological Agency. Thus, the METEX model is proper to examine short-distance vector dispersal between islands.

#### Evaluation of vector dispersal by wind between the islands

**Fig. 1:**
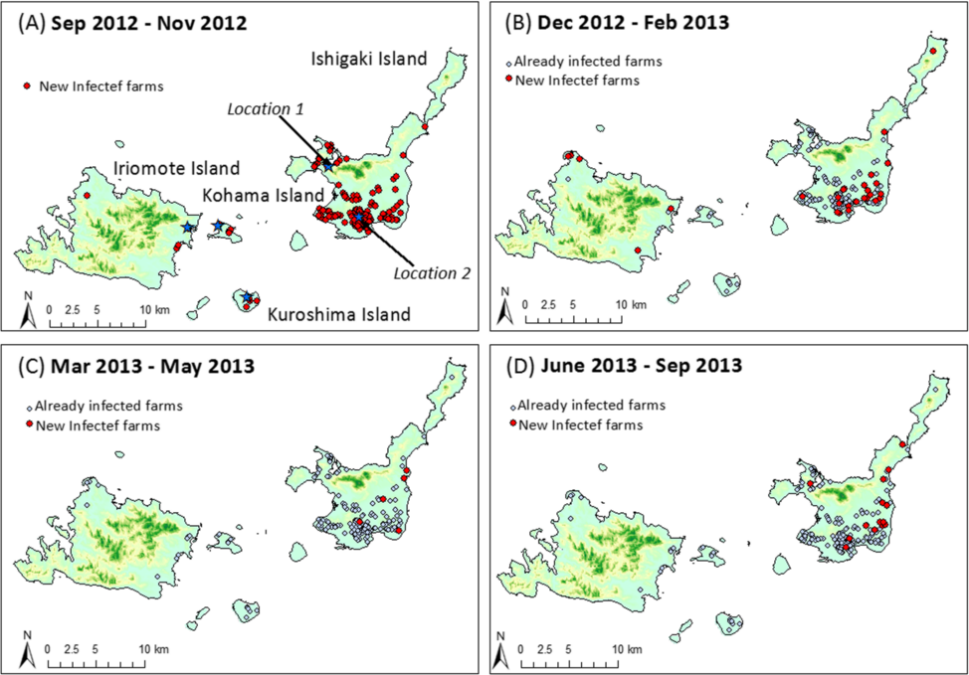
Distribution of infected farms in the Yaeyama Islands during the BEF outbreak in 2012–2013. The blue stars indicate the first case of BEF on each island and the starting points of the trajectory (on Location 1 and Location 2). The red dots show new infected farms during the period September 2012 to November 2012 (**a**), December 2012 to February 2013 (**b**), March 2013 to May 2013 (**c**), and June 2013 to September 2013 (**d**)

The possibility of windborne transmission of vectors from Ishigaki Island to surrounding islands was examined by forward trajectory analysis using the METEX model. Because of the spread of the disease in Ishigaki Island, two sites were set as the starting points of the trajectory: the first site was where the first case of BEF occurred in the western part of Ishigaki Island (i.e., Location 1) and the second site was where the first case of BEF occurred in the southern part of Ishigaki Island (i.e., Location 2), which was a major epidemic area with a high density of cattle farms (Fig. 1).

To examine the possibility of windborne transmission of vectors between the islands, the terms “potential source period” and “incursion risk period” were defined as follows:

##### Potential source period

The period in which infected vectors have already invaded in an area and the infected vectors can disperse into other areas. After BEF is first detected in an area, it is assumed to be in the potential source period. This period is assumed to continue at the end of the epidemic because of the possibility that infected vectors survive in the area.

##### Incursion risk period

The period during which the incursion event is most likely to have occurred. The duration of this period was set 14 days before the detection of the first case of BEF in an area. This period was based on the incubation period of the BEFV (usually 2–4 days with a maximum of 10–11 days) [2, 46], and on the estimated lifespan of adult insects in the field (1–2 weeks for mosquitoes and *Culicoides* biting midges) [31, 47, 48].

Based on these definitions, the potential source period of Location 1 and Location 2 on Ishigaki Island was determined as beginning on September 10, 2012 and September 16 2012, respectively (Table 1). The incursion risk period of each island was September 1–14, 2012 for Iriomote and Kohama Islands and September 12–25, 2012 for Kuroshima Island.

**Table 1:** First notification date, potential source period, and incursion risk period of each island

Island	First notification date	Potential source period	Incursion risk period
Ishigaki			
Location 1	Sep 10 2012	After Sep 10 2012	Aug 27–Sep 9 2012
Location 2	Sep 16 2012	After Sep 16 2012	—
Kohama	Sep 15 2012	—	Sep 1–14 2012
Iriomote	Sep 15 2012	—	Sep 1–14 2012
Kuroshima	Sep 26 2012	—	Sep 12–25 2012

Using the METEX model, 24-h forward trajectories were generated at hourly intervals from Location 1 and Location 2, and the likely dates of windborne transmission of vectors to the surrounding islands within the incursion risk period were explored.

#### Estimation of the potential sources of the vectors

Potential sources of the infected vectors were estimated by backward trajectory analysis using the HYSPLIT model. The location of the first case in Ishigaki Island (i.e., Location 1) was set as the starting point of the trajectory. During the incursion risk period in Location 1 (August 27, 2012–September 9, 2012), 72-h back trajectories were generated at hourly intervals. The length of the trajectory was based on the assumption that the vectors travelled the approximately 2000-km distance between Southeast Asia and Ishigaki Island in 3 days. With regard to the longevity of the vectors, female mosquitoes and *Culicoides* biting midges reportedly survive 3–5 days without sugar or a blood meal [49–51]; therefore, the 72 h-length of the trajectory is supposed to represent the possible situation of infected vectors reaching the islands through a windborne flight across the sea.

### Spatial analysis

#### Data

Spatial statistical modeling was conducted to explore the factors associated with vector transmission within Ishigaki Island. The incidence risk on 1-km grid cells of the island, which was defined as the proportion of the cumulative number on infected farms to the total number of cattle farms within the grid cell, was analyzed as the dependent variable. The terrain and land use data were analyzed as the explanatory variable. The grid cell size was determined by taking into account the spatial dependency of infected and noninfected farm distribution. This spatial dependency of farm distribution was checked by a semivariogram, which defines the spatial scales over which the pattern are dependent [52]. In plotting a semivariogram, a saturated state indicates a pattern that lacks special dependencies [52]. In this study, the semivariograms were calculated by a farm’s status (i.e., 0, noninfected; 1, infected) and by the distance between farms. Visual inspection of the semivariogram plots detected a saturated pattern of semivariogram when the distance was greater than 0.8 km. Thus, the 1-km grid cell size was considered an appropriate size to exclude farm spatial dependency.

Environmental information such as elevations and slope gradients, land use information, and farm density (i.e., the number of cattle farms in a 1-km grid cell) was selected as the potential predictors of disease transmission on the island. Data on the elevations and slope gradients in each 1-km cell were obtained from a free publicly accessible database, the National Land Numerical Information (NLNI) by the Ministry of Land, Infrastructure, Transport and Tourism (MLIT) in Tokyo, Japan [53]. The average value, minimum value, and maximum value of the evaluations and slope gradients in each cell were used for the analysis. The 100-m grid cell sized land use data, obtained from the NLNI [53], were used to calculate the proportion of each land use classification for each 1-km cell. Land use was classified as paddy fields; farmland (except for paddy fields), which mostly included sugar cane and pasture in the island; forest; wasteland; building use; inland water (i.e., rivers and lakes); and others.

### Statistical analysis

The number of BEF cases and the number of farms in a cell *i* are *y*_*i*_ and *n*_*i*_, respectively; therefore, the expected incidence risk, *p*_*i*_, in cell *i* can be described by the following logistic regression formula:

$$ \begin{array}{l}{y}_i\sim Bino \min al\left({p}_i{n}_i\right)\hfill \\ {} \log it\left({p}_i\right)= \log \left(\frac{p_i}{1-{p}_i}\right)={\beta}_0+Z\beta +\varepsilon \hfill \end{array} $$

in which *β*_*0*_ represents the intercept, *Zβ* represents a series of explanatory variables, and *ε* represents the residual term (assumed to have an approximately normal distribution).

In spatial analysis, unknown factors often vary in space and induce a spatial correlation between the observed disease counts in each area and its neighbors [54]. To account for this correlation, it is assumed that the unexplained variation comprises two parts: a structured component (i.e., spatially correlated) and an unstructured component (i.e., spatially random). In this context, the aforementioned model was parameterized as follows:

$$ \log it\left({p}_i\right)= \log \left(\frac{p_i}{1-{p}_i}\right)={\beta}_0+Z\beta +{S}_i+{U}_i $$

in which *S*_*i*_ and *U*_*i*_ correspond to the structured and unstructured random effects, respectively, for cell *i*,. The unstructured heterogeneity term, *U*_*i*_, was assumed to have a normal distribution with mean 0 and precision (inverse variance) *τ*_*u*_. The structured heterogeneity term, *S*_*i*_, was assumed to have a conditional intrinsic Gaussian autoregressive (CAR) structure, which was described in 1991 by Besag et al. [55]. It has been applied in previous spatial modeling studies in epidemiology and ecology [56–59]. The inclusion of the CAR structure allows the incidence risk of cell *i* to be modeled by taking into account the spatial structured effects from cell *i*’s neighbors. In this paper, we specified that cell *j* is a neighbor of cell *i*, if they share the same boundaries. In the CAR structure, the spatial component *S*_*i*_ is assumed to depend on a conditional distribution that is a normal distribution with a mean equal to the average of the spatial component in the set of cells (i.e., neighbors) adjacent to cell *i* and precision proportional to the number of neighbors (this precision is defined as *τ*_*s*_). Non-informative prior distributions were given for the priors of the intercept and coefficients of explanatory variables, assuming normal distribution with a mean of 0 and a variance of 10^−5^. The precisions of the unstructured and structured spatial components were also given non-informative distribution by gamma distribution (shape, 0.5; scale, 0.0005) [60].

In the spatial analysis, all explanatory variables were first subjected to univariable logistic regression. Only variables with a *p* value less than 0.15 in the univariable analysis were then included in the multivariable logistic regression model. In multivariable analysis, all possible combinations of explanatory variables were examined, and the model with the smallest Akaike information criterion (AIC) was determined as the best-fitting model without taking into consideration spatial correlation [61]. Finally, the explanatory variables selected in multivariable analysis were included for the CAR model, which took into consideration the unstructured and structured spatial components (Model 1). To evaluate the influences of unstructured and structured random effects, we also compared three other models: Model 2, which included only the structured random effect; Model 3, which included only the unstructured random effect; and Model 4, which did not include the structured random effect or the unstructured random effect. Spatial autocorrelations of residuals in these models were assessed using Moran’s *I* index [62] and the goodness of fit for the models was compared using the deviance information criterion (DIC). These hierarchal spatial models were run by the Markov chain Monte Carlo algorithm implemented Bayesian Inference Using Gibbs Sampling software (WinBUGS 1.4) [63]. R2WinBUGS package [64] and R version 3.10 (R Core Team, 2014) were used to run the model in WinBUGS. In the model, three chains were simulated, and each chain had 500,000 iterations after a burn-in of 20,000 iterations. After storing every 10th iteration from each chain, 150,000 iterations were used for the posterior parameter estimates. The explanatory variables were scaled to a mean of zero and standard deviation of one prior to the model to facilitate the convergence of model estimate. Convergence was assessed by the visual inspection of the plots of the sampled parameters and values of R-hat by the Gelman-Rubin convergence diagnostic, which indicate convergence when the values are close to 1 [65].

## Results

### Descriptive analysis and transmission between islands

**Fig. 2:**
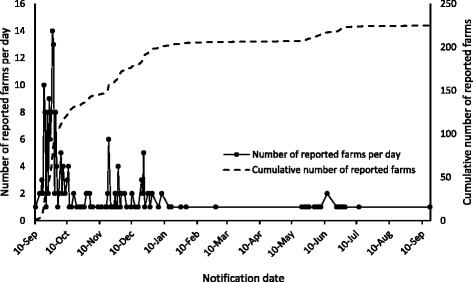
The number of infected farms during the bovine ephemeral fever outbreak in 2012–2013

The first case of BEF was detected on a beef-breeding farm in the western part of Ishigaki Island on September 10, 2012 (Figs. 1 and 2). By the middle of September, the disease had spread to the southern part of Ishigaki Island; it was also confirmed on Iriomote and Kohama Islands on September 15, 2012. The disease was then confirmed on Kuroshima Island on September 26, 2012. Between October 2012 and January 2013, BEF cases were detected sporadically in the southern and eastern parts of Ishigaki Island. The last case of BEF was reported on Ishigaki Island on September 17, 2013. Further suspected cases have not been observed. All 225 cases of BEF were detected on beef cattle farms. The farm-level incidence risk on each island was the following: Ishigaki Island, 39 % (204/523, 95 % confidence interval [CI], 35 %–43 %), Iriomote Island, 20 % (10/49, 95 % CI, 9 %–32 %); Kohama Island, 19 % (5/26, 95 % CI, 4 %–34 %); and Kuroshima Island, 11 % (6/55, 95 % CI, 3 %–19 %).

**Fig. 3:**
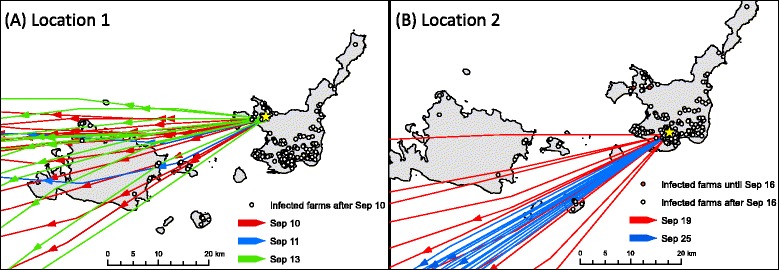
Forward trajectories from Ishigaki Island to neighboring islands. The yellow stars indicate the starting point of trajectory, (i.e., Location 1 and Location 2) on Ishigaki Island

The distributions of forward trajectories that originated from Ishigaki Island are shown in Fig. 3. During the potential source period of Location 1 on Ishigaki Island, dispersal by air to Iriomote and Kohama Islands occurred on September 10, 11, and 13—dates that were within the incursion risk period of these islands. In addition, during the potential source period of Location 2 on Ishigaki Island, dispersal by air to Kuroshima Island occurred on September 19 and September 25, which was within the incursion risk period on Kurohama Island. Wind dispersal from Ishigaki Island occurred 1 week before the first detection of the disease on each island. Except for these wind events, no typical wind blew from Ishigaki Islands to the surrounding islands within the incursion risk period of each island.

### Estimation of the potential source of the vectors

**Fig. 4:**
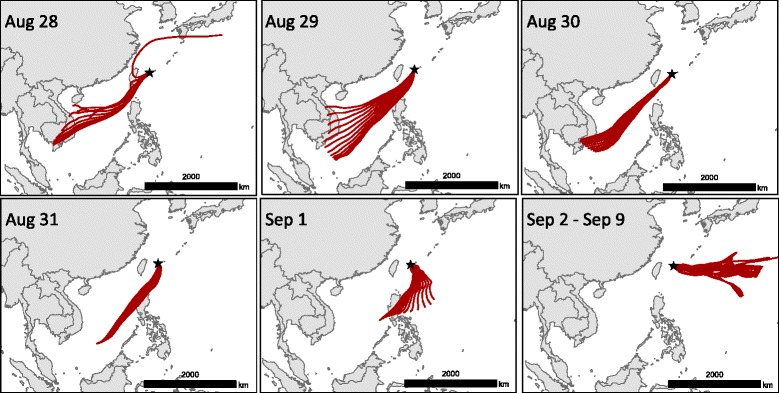
Backward trajectories from Ishigaki Island. The black star indicates the starting point of the trajectory at Location 1 on Ishigaki Island

Results of backward trajectory analysis showed that the source of trajectories reaching Ishigaki Island were Viet Nam, Laos, and the Philippines during the incursion risk period (August 28, 2012–September 1, 2012); a few trajectories were observed from Taiwan on August 28, 2012 (Fig. 4). However, during September 2–9, 2012 (i.e., 1 week before the first detection of the disease), no back trajectories extended to Southeast Asia, and the terminal points of the trajectories were distributed over the sea to the east coast of the island (Fig. 4).

### Spatial analysis

**Fig. 5:**
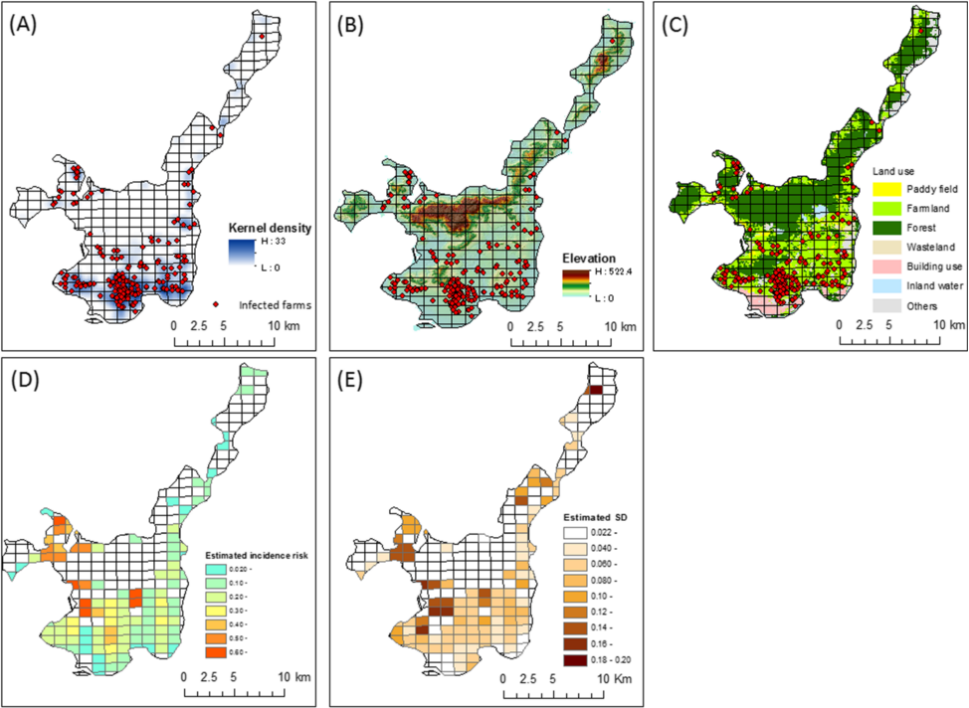
The spatial analysis results. **a** The distribution of infected farms and kernel density of cattle farms on Ishigaki Island. **b** Elevation and topography of Ishigaki Island. **c** Land use on Ishigaki Island. **d** The estimated incidence risk in each 1-km cell. **e** The estimated standard deviations of each 1-km cell

The distribution of infected farms with the kernel density of cattle farms, elevation and topography and the land use of the island is shown in Fig. 5a–c, respectively. There were 122 grid cells, which included cattle farms. The median number of cattle farms per cell was four farms (range, 1–33 farms). As a result of univariable and multivariable analyses, the 1-km cell-level incidence risk was best explained by the model, which included the minimum slope gradient (*p* = 0.01), the proportion of paddy fields (*p* = 0.01), and the proportion of farmlands (*p* < 0.01). The results of the assessment of residuals and the goodness of fit for the hierarchal spatial models, which include these three variables, are shown in Table 2. In Models 3 and 4, which did not include the structure random effect, autocorrelations of residuals were marginally detected (*p* = 0.07) and the values of DIC were larger than those of Models 1 and 2, which included the structure random effect. Among the four models, Model 2 gave a better fit in terms of a lower DIC value; thus, the results of Model 2 were used for the final analysis (Table 3). The posterior distribution of parameters in the model was converged (i.e., the value of R-hat was nearly equal to 1). With regard to the factor of land use, the proportion of paddy fields and the proportion of farmlands showed a positive impact on the 1-km cell-level incidence risk, although the proportion of paddy fields was a little less than 0 in the 2.5 % credible value, which was significant in the multivariable logistic mode. With regard to the terrain factor, the minimum slope gradient was a significant predictor for the 1-km cell-level incidence risk (i.e., a cell with a lager minimum slope gradients tended to have a higher incidence risk). The predicted 1-km cell-level incidence risk and its estimated standard deviation are shown Fig. 5d, e. Relatively high incidence risk areas were predicted in the western to southwestern region of Ishigaki Island. In some grid cells with a small number of cattle farms (i.e., fewer than five farms), the estimated standard deviations of predicted incidence risk were a little higher than in the other grid cells.

**Table 2:** Moran’s *I* index for residuals and the deviance information criterion of four hierarchical spatial models

Models	Parameters^a^	Moran’s *I* (p value)	DIC
Model 1	Fixed effects, S, U	−0.1031 (0.9157)	306.385
Model 2	Fixed effects, S	−0.1193 (0.9467)	301.918
Model 3	Fixed effects, U	0.0923 (0.0721)	323.569
Model 4	Fixed effects	0.0888 (0.0794)	331.492

*DIC* deviance information criterion

^a^Fixed effects indicate variables that were significant in univariable and multivariable analyses and included in the hierarchical spatial models. The letters “S” and “U” indicate the structured and unstructured heterogeneity term, respectively

**Table 3:** The posterior means and standard deviations of the regression coefficients in Model 2

Variables	Mean	SD	95 % CI	R-hat
Minimum slope gradients^a^	0.2941	0.1453	0.0049, 0.5777	1.0459
Proportion of paddy fields^a^	0.1556	0.1493	−0.1320, 0.4565	1.0074
Proportion of farmland^a^	0.4603	0.1413	0.1863, 0.7469	1.0341
Structured heterogeneity term^b^	0.0014	0.0008	0.0002, 0.9042	1.1295

Model 2 is the hierarchical spatial model which included structured heterogeneity term. 95 % CI, 95 % credible interval; *SD* standard deviation

^a^Each estimated regression coefficient is the standardized coefficient, which indicates the relative contribution to the incidence risk of bovine ephemeral fever

^b^The standard deviation of the structured heterogeneity term

## Discussion

The present epidemiological analysis clarified the spatial and temporal characteristics of the 2012–2013 BEF outbreak in the Yaeyama Islands. The first BEF case was detected on a beef cattle farm in the western part of Ishigaki Island. In 1 month, the disease had spread to the southern part of the island and was transmitted to neighboring islands. Analysis using an atmospheric dispersion model showed that the forward trajectories from Ishigaki Island dispersed to the neighboring islands 1 week before the disease was detected on each island. This result suggested that wind dispersion of infected vectors could occur from Ishigaki Island to neighboring islands during the high risk incursion period for each island. The potential of introducing BEF *via* infected cattle movements from Ishigaki Island to neighboring islands was negligible, based on the cattle movement data during the early phase of the outbreak. Furthermore, the introduction of infected cattle from surrounding Asian countries was negligible because the importation of cloven-hoofed animals and their products are prohibited from these countries on account of the outbreaks of foot-and-mouth disease. Therefore, atmospheric transport of infected vectors is presumable in the transmission of the disease between islands.

The windborne spread of bluetongue virus infection and Schmallenberg virus infection has also been reported in the recent emergence of these diseases in European countries [18–23, 66]. In the United Kingdom and Sweden, analysis using atmospheric dispersion models well demonstrates the possibility that infected vectors were likely to have reached over the sea from affected regions in Europe [19, 20]. In comparison to the transmission distance in Europe (approximately 100–200 km), the distance between Ishigaki Island and neighboring islands are extremely short (approximately 15 km); thus between-island transmission of infected vectors *via* wind could have readily occurred.

The BEF outbreak continued in Ishigaki Island for 1 year, which included the winter. The BEFV gene from blood samples from febrile cattle in April 2013 shared the identical sequence of the virus isolated in 2012. In infected cattle, the viremic period of BEFV usually lasts 4–5 days [67]; therefore, it is probable that the virus had been maintained in a cycle between the vector and the host, even in the winter. The winter season in Yaeyama Island is milder and shorter (average temperature is approximately 20 °C); as a result, the maintenance of BEFV would be possible, even in the winter.

The influence of landscape on an outbreak of arbovirus infection or the abundance of the related vectors has been investigated in previous studies. The abundance of *Culicoides* biting midges is positively associated with pasture cover [32]. Woodland, open prairies, and impervious surface areas have been associated with the risk of bluetongue infection [36]. Furthermore, row crops, shrublands, soil, and wetlands are positively associated with West Nile virus transmission by mosquitoes [37]. The BEFV has been isolated from field-caught mosquitoes and biting midges [2, 67]. In addition, viral replication is also evident in both insects by experimental feeding on infected blood [2, 67]. In the present study, spatial analysis showed that the proportion of paddy fields and farmlands was significantly associated with the incidence risk within Ishigaki Island. Biting midges and mosquitoes require wet soil or semiaquatic habitats for breeding; therefore, the areas covered with paddy fields or farmlands are suitable for the growth and survival of the vectors. A previous field study in Japan demonstrated that the larvae of some species of *Culicoides* biting midges such as *C. oxystoma*, which has a high potential to transmit ruminant arboviruses [68], were recovered from paddy fields. This finding suggests that paddy fields contribute to the breeding and maintenance of biting midges [69]. Furthermore, it is important to note that farmlands on Ishigaki Island are primarily used for pastures and sugar cane. Larvae of *C. asiana*, which is a recently recognized cryptic species of *C. brevitarsis* [70, 71]*,* have been collected from cattle dung in pastures in the Yaeyama Islands in a previous field survey [69]. Previous entomological surveillance in Ishigaki Island also shows that these species are dominant or subdominant in cowsheds [72, 73]. Meanwhile, the larvae of Anopheline and Culicine mosquitoes are frequently collected from paddy fields in the Yaeyama Islands [74, 75]. Several species such as *Anopheles sinensis* and *Culex tritaeniorhynchus*, which breed in paddy fields, obtain their blood meal from cattle [76]. If one or more of these hematophagous insects contribute to the transmission of BEFV, paddy fields and farmlands could be a high risk factor for disease spread. Little information on the vectors of BEF has been available in this region; however, the spatial analysis in this study showed that farmlands and paddy fields would be logical search areas if studies were conducted to identify BEF vectors in this area, and it would support an understanding of the ecological mechanism of BEFV circulation.

Spatial analysis also showed that the explanatory variable of the minimum slope gradient was positively associated with the incidence risk. This result indicates that a sloping area tended to have a higher incidence risk. In general, the nature of air flow is complex and a variety of local winds arise in complex terrains such as a hilly or mountainous terrain [14, 77]. Local winds may influence the movement and concentration of flying insects [14, 77]. Some insects such as grasshoppers or moths move up and down the slopes by using the updraft or downdraft from the terrains; in addition, rotor waves or lee waves deposit insects in an area and produce localized outbreaks of a pest species [14, 77–79]. The western part of Ishigaki Island is a mountainous region; therefore, the complicated condition of its terrain may concentrate the vectors and lead to a BEF outbreak.

The backward trajectory analysis in this study estimated the possible source of infected vectors in Southeast Asian regions such as Viet Nam, Laos, and the Philippines. Mitochondrial phylogeography analysis reveals that *C. oxystoma* specimens captured in Japan is closely related to specimens captured in Southeast Asia [80]. A few backward trajectories from Taiwan were also observed, which suggested that Taiwan was not negligible as a potential source. A previous study demonstrated that the Japanese and Taiwanese strains of the BEFV, which were isolated in the outbreaks of 1996, 1984–1989, and 1996–2004, were classified in the same clusters and were closely related, based on their nucleotide sequences [10]. As in Japan in 2012, an outbreak of BEF was reported in Taiwan, which is adjacent to the Yaeyama Islands [13]. The BEFV isolated in the 2012 outbreak was classified in the same cluster [9]. With regard to other arbovirus infections, Japanese encephalitis virus, which is transmitted by mosquitoes, was isolated from pigs on Ishigaki Island and was most closely related to the Taiwanese strains [81]. These findings support that the arbovirus infections in Japan and Taiwan have an epidemiological linkage and are exposed from the enzootic lower latitude regions *via* long-distance movements of infected vectors carried by wind. Rice planthoppers, a major rice pest in Japan, can fly more than 2000 km by wind from Southeast Asia or from southern China to Japan [82]. Therefore, the windborne long-distance migration of BEF-infected vectors would be possible, even for *Culicoides* biting midges (1–3 mm), which are smaller than rice planthoppers (4–6 mm). Overseas migration of *Culex* mosquitoes, which are the vector for the Japanese encephalitis virus, has also been suggested in Asia and Oceania [83–85]. The possibility of BEF introduction by the infected mosquitoes from lower latitude regions could not be ruled out at present.

This study clarified the spatiotemporal characteristics of a BEF outbreak in Japan and revealed the risk factors associated with the disease transmission in Ishigaki Island. However, because of the following limitations of this study, some degree of caution is needed in interpreting these results. First, the outbreak data used for the analysis in this study focused on BEF cases that were based on clinical and epidemiological observations. Because of the possibility of subclinical infection, the disease may have in actuality spread wider. Second, the present analysis did not take into account the effect of vaccination. After the outbreak of BEF, emergency vaccinations with a single dose of inactivated vaccine were administered to the cattle in the region; vaccination was especially mandatory for cattle that were shipped to market and 8182 cattle (24 % of cattle in the region) were vaccinated. However, because serological surveillance had not been conducted, we did not explore the effect of vaccination in the current study. The routine vaccination of dairy cattle and beef heifers has been also encouraged in the Yaeyama region as a preventive measure of BEF. However, the vaccination coverage of all cattle population in this region was low in past 3 years: the vaccine coverage and the number of vaccinated animals were 2.6 % and 875 animals in 2010, 2.5 % and 808 animals in 2011, and 1.3 % and 410 animals in 2012 (except for emergency vaccination). Therefore, the influence of vaccination in this region seems to have a limited effect on BEF infection.

In this study, the potential sources of infected vectors were estimated in Southeast Asia. The prediction of the migration of infected vectors to Japan will be useful to prevent and control BEF. However, further investigations are still needed to achieve this. First, although BEF is a disease that occurs in tropical, subtropical, and temperate regions in Asia [2], the current situation of BEF in Southeast Asia has not been investigated well. With the evidence that BEF has been confirmed in the southeast region of China [12, 86], it is highly probable that the disease is enzootic in Southeast Asia. Second, the air temperature at flying height was not considered in the trajectory analysis. To refine the prediction of infected vector immigration, more detailed information on meteorological conditions such as temperature, humidity, and wind speed would need to be considered. In Japan, the migration simulation model for rice planthoppers has been developed by incorporating a numerical weather prediction model and a particle dispersion model, and it has been put to practical use [87, 88]. This approach would be a useful reference to predict the migration of infected vectors in relation to BEF. Third, the prediction of infected vector migration is difficult to validate. As discussed in a previous study on the dispersal model of *Culicoides* biting midges in Australia [24], areal collections, use of entomological radars, or recapture technics are not feasible for biting midges because of its smallness and fragileness. Therefore, a mitochondrial genome phylogeographic analysis has been established to distinguish between invasive and indigenous species of biting midges [80]. A further knowledge and understanding of these factors will refine the prediction of migrating infected vectors.

## Conclusions

The epidemiological analysis in this study demonstrated that the condition of topography and landscape had a significant influence on the spatial distribution of BEF within Ishigaki Island. There is a complex interaction between hosts, vectors, and viruses in arbovirus infection outbreaks. Therefore, the landscape components that affect the distribution of vectors and their habitats would be important when assessing the favorable environments for arbovirus infections such as BEF. Furthermore, trajectory analysis using atmospheric dispersion models demonstrated that the long-distance migration of infected vectors would have occurred from Southeast Asia and that the local-scale between-island transmission by wind would have occurred during the outbreak. These findings would be helpful in developing a surveillance program and preventive measures against BEF.

## Abbreviations

AIC: Akaike information criterion
BEF: bovine ephemeral fever
CAR: conditional intrinsic Gaussian autoregressive
CGER: Centre for Global Environmental Research
DIC: deviance information criterion.
GDAS: global data assimilation system
HYSPLIT: hybrid single particle lagrangian integrated trajectory model
METEX: Meteorological Data Explorer
MLIT: Ministry of Land, Infrastructure, Transport and Tourism
NLNI: National Land Numerical Information
